# MicroRNA-142-3p Inhibits Tumorigenesis of Colorectal Cancer *via* Suppressing the Activation of Wnt Signaling by Directly Targeting to β-Catenin

**DOI:** 10.3389/fonc.2020.552944

**Published:** 2021-02-10

**Authors:** Peng Liu, Fuao Cao, Jinke Sui, Yonggang Hong, Qizhi Liu, XianHua Gao, Haifeng Gong, Liqiang Hao, Zheng Lou, Wei Zhang

**Affiliations:** Colorectal Surgery Department, Changhai Hospital, Naval Medical University, Shanghai, China

**Keywords:** microRNA-142-3p, colorectal cancer, Wnt signaling, β-catenin, CTNNB1

## Abstract

**Background:**

Altered expression profile of microRNAs (miRNAs) was reported to be associated with colorectal cancer (CRC). The aims of this study are to identify the changed miRNAs in the plasma of CRC patients and explore the underlying mechanism of these miRNAs during tumorigenesis.

**Methods:**

Plasma miRNA expression profiles were compared between healthy people and CRC patients. MiRNA expression was measured using quantitative real-time PCR. Colony formation and MTT assays were used to test cell proliferation. Luciferase assay, immunohistochemistry and Western blotting were employed to explore the molecular mechanism.

**Results:**

MiR-142-3p level was found as the most significantly repressed miRNA in CRC patients. Overexpression of miR-142-3p dramatically repressed colony formation and cell proliferation of both HT29 and HCT116 cells while inhibition of miR-142-3p promoted those of the cells. Interestingly, overexpression of miR-142-3p reduced the level and nuclear accumulation of β-catenin. We further observed that miR-142-3p remarkably inhibited the transcriptional activity of β-catenin gene (CTNNB1). However, mutations in the predicted binding sites blocked this inhibition, suggesting that miR-142-3p may directly bind to the mRNA of β-catenin.

**Conclusion:**

In conclusion, we identified miR-142-3p exerts its function as a tumor suppressor through blocking the activation of Wnt signaling by directly targeting to CTNNB1.

## Introduction

Colorectal cancer (CRC) has become a severe health burden due to its high morbidity and mortality world-wide (1). Both inherited and sporadic mutations contribute to the development of CRC, which could be commonly detected by biopsies. Although nowadays, earlier detection and various treatments have shown positive effects on improvement of survival rate, still about half of the patients die from CRC mainly due to the occurrence of metastasis.

Surgical removal is a common treatment that may be combined with chemotherapy and/or targeted biologics for patients with late-stage of CRC. There are a number of novel biologics that target essential factors for tumorigenesis including the Wnt signaling (2) and epidermal growth factor receptor (EGFR) (3). It is well known that Wnt signaling is essential for the proliferation and maintenance of intestinal stem cells which locate at the bottom of the crypts and are capable of differentiating into numerous cell types (4). Dysregulation of Wnt signaling contributes to various cancers, especially CRC. The Wnt signaling is activated by dissociation of β-catenin from the destruction complex composed of adenomatous polyposis coli (APC), AXIN1, casein kinase 1 (CK1), and glycogen synthase kinase 3β (GSK3β). Stabilized β-catenin translocates to nuclear and further binds to TCF/LEF for transcription initiation (5). A set of evidence demonstrated that hyperactivity of Wnt plays a key role as an oncogenic driver in CRC (6). APC mutation is found to be a major contributor to CRC development whereas only a small number of CRC cases were found to have AXIN and β-catenin mutations. However, to date, none approved drugs targeting Wnt signaling are available in the clinic, which is mainly caused by its important roles in the normal developmental and biological activities as well as its complex interactions with other cellular pathways. Other approved drugs such as EGFR inhibitors showed limited successful responses from patients (3). These challenges gave rise to the needs for identifications of new targets.

MicroRNAs (miRNAs) belong to a family of small noncoding RNAs that comprises 18 to 25 nucleotides, which bind to the 3′ untranslated region (UTR) of their target mRNA. They exert their functions as inhibiting gene expression or triggering mRNA degradation. Therefore, they control various biological activities of cells including cell proliferation, differentiation and apoptosis (7). Currently, increasing evidence have shown the alterations of miRNA expression in CRC by analyzing the miRNAs expression profiles. For example, a research reviewed 23 studies and concluded that more than 100 miRNAs were upregulated whereas about 50 miRNAs were downregulated in CRC (8). Recently, miR-142-3p expression was identified to be highly associated with CRC and may affect cell proliferation by targeting TCF7 (9). We found that the plasma miR-142-3p levels in CRC patients were remarkably decreased compared to those of healthy people in this study. We observed that upregulation of miR-142-3p led to a significant decrease of cell growth and colony formation. To explore the underlying mechanism, we explored its effects on Wnt signaling and identified β-catenin as a direct target of miR-142-3p.

## Materials and Methods

### Cell Culture

A human colon fibroblasts cell line CCD-18Co and four CRC cell lines including HT29, SW480, HCT116, and Caco-2 were purchased from American Type Culture Collection (ATCC, Rockville, MD). CCD-18Co cells were maintained in Dulbecco’s Modified Eagle Medium (DMEM) with high glucose supplemented with 10% to 20% fetal bovine serum (FBS, Invitrogen, Carlsbad, CA, USA), 1% sodium pyruvate (100 mM), 1% non-essential amino acids (10 mM), and 1% penicillin/streptomycin. 1% sodium pyruvate (100 mM). HT29 and HCT116 cells used McCoy’s-5a–modified medium (Gibco, Grand Island, NY, USA), SW480 used RPMI 1640 medium (Invitrogen, Carlsbad, CA) and Caco-2 used Leibovitz’s L-15 Medium (Gibco), supplemented with 10% to 20% of FBS and 1% penicillin/streptomycin. Cells were maintained at 37°C with 5% CO_2_ and humidified air.

### MiRNA Profiling Using miRNA Microarray and Quantitative Real-time PCR

To compare the miRNAs profiles, we performed miRNA microarray. RNA from plasma was extracted using mini miRNeasy kit from Qiagen (Venlo, Holland) according to the manufacturer’s instruction. The samples were sent to the Kangchen Biotechnology company (Shanghai, China) for testing using GeneChip miRNA 2.0 Array (Affymetrix, Santa Clara, CA, USA). For quantitative real-time PCR (qRT-PCR), the expression levels of the miRNAs were evaluated using TaqMan MicroRNA Assays kit (Ambion) according to the manufacturer’s instructions. The results were measured by the 2^−ΔΔ^Ct method. All values were normalized to RNU6B (Ambion).

### Plasmids and Transfection

Overexpression and inhibition of miR-142-3p were achieved by transfection of cells with synthetic miRNA mimic or inhibitor. For the luciferase assay, the predicted binding sites on β-Catenin 3′-UTR from 406 to 412 or 763 to 770 bp were cloned into a pGL3-basic luciferase reporter plasmid (Promega, Madison, WI, USA). All transfections were performed using Lipofectamine 2000 reagent (Invitrogen, Carlsbad, CA, USA). The procedure was conducted according to the manufacturer’s instructions.

### Colony Formation and Cell Proliferation Assays

For the colony-formation assay, cells (500 to 1,000 cells per well) were seeded into six-well plates and cultured for 14 days. The colonies were fixed and stained with Giemsa. Colony numbers were counted and triplicate wells were counted for each group.

For MTT assay, cells (2,000 to 3,000 cells per well) were seeded into 96-well plates. Cell Proliferation Reagent Kit I (MTT; Roche Applied Science) was used for testing the cell proliferation each day. The results were read by absorbance at 490 nm using a plate reader (BioTek Company).

### Western Blotting

Proteins were prepared by lysis buffer (20 mM Tris HCl pH 8, 137 mM NaCl, 1%, Nonidet P-40 (NP-40), and 2 mM EDTA) or Cell Fractionation Kit (CST#9038, Cell Signaling Technology, Danvers, MA, USA). The proteins were separated using 10% sodium dodecyl sulfate polyacrylamide gel electrophoresis (SDS-PAGE) and transferred onto NC membranes (0.22 μm, Sigma-Aldrich, St. Louis, MO, USA). To block the membranes, 10% nonfat milk was used. Primary antibodies including β-catenin (CST#8480), α-tubulin (CST#2125), and histone H3 (CST#4499) were applied. The membranes were washed three times by tris-buffered saline with 0.1% Tween 20 (TBST). Horseradish peroxidase (HRP)-conjugated secondary antibody (Santa Cruz, CA, USA) was used and the signals were detected using an ECL kit (Thermo Fisher Scientific, Waltham, MA, USA).

### Immunocytochemistry and Immunohistochemistry

Immunocytochemistry of HT29 cells was performed. Briefly, cells were collected by trypsin and fixed with fresh 4% paraformaldehyde. After acid denaturation, cells were immunostained with anti–β-catenin (Millipore; Billerica, MA, USA). Elite Vector Stain ABC systems (Vector Laboratories) were used for the reactions. DAB substrate chromogen (DakoCytomation) followed by hematoxylin counterstaining were performed for visualization. For tumor samples, tumors were fixed in 4% paraformaldehyde overnight and proceeded paraffin-embedded. Sections were immunostained with anti–β-catenin (Millipore).

### Luciferase Assay

HT29 cells stably expressing miR-142-3p mimics or inhibitors were seeded in triplicate in 24-well plates and cultured for 24 h. Then, luciferase reporter plasmids (100 ng) and pRL-TK Renilla plasmid (10 ng, Promega) were co-transfected into the cells. The signals were detected using a Dual-Luciferase Report Assay Kit (Promega) after 24 h of transfection.

### Xenograft Mouse Model

Tumorigenesis was evaluated by a xenograft mouse model. Cells (1 × 10^6^) transfected with miR-142-3p mimics or inhibitors were subcutaneously injected into 6-week-old nude mice obtained from Shanghai Laboratory Animal Center (SLAC, Shanghai, China). All animal experiments were approved by Changhai Hospital Ethics Committee (2017–029). Tumor volumes were evaluated and recorded at indicated days (days 15, 18, 21, 24, 37, 30) using the formula 0.44× diameter of the tumor × perpendicular value^2^.

### Statistical Analysis

All values were stated as mean± standard deviation (S.D.) or frequencies and percentages. SPSS (version 16.0, IBM Corporation, Armonk, NY, USA) was used for statistical analysis. T-tests were used for the determination of statistical significance. A p value less than 0.05 was considered as significant.

## Results

### MiR-142-3p Expression Is Reduced in the Plasma of Patients with Colorectal Tumor and Colorectal Cancer Cell Lines

**Figure 1:**
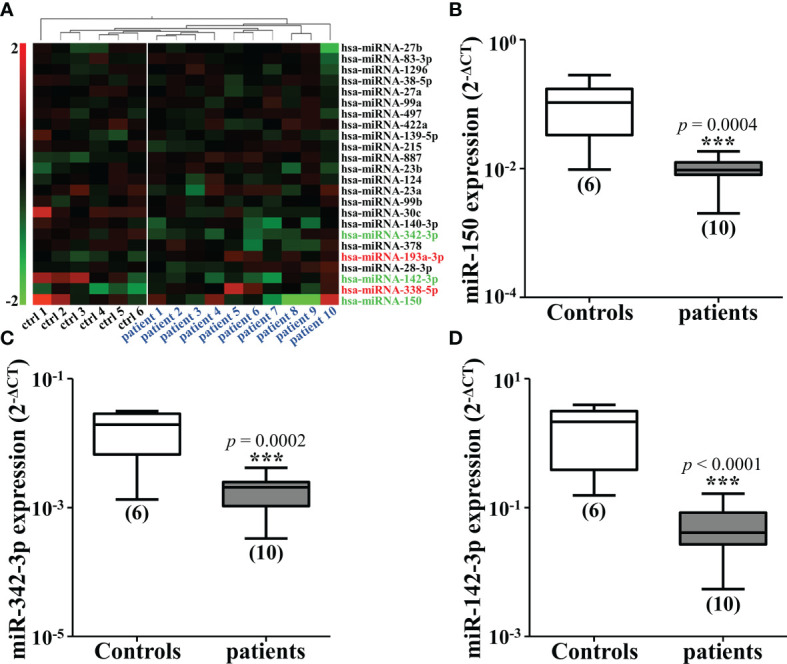
Mi-142-3p is down-regulated in the plasma of patients with colorectal tumor. **(A)** Cluster analysis of miRNAs in the plasma of 10 CRC patients and 6 healthy controls. The expressions of down-regulated miRNAs, including miR-150 **(B)**, miR-342-3p **(C)** and miR-142-3p **(D)** in the plasma of CRC patients and healthy controls were detected by qRT-PCR. All data are shown as means ± SD. ***P<0.001.

**Figure 2:**
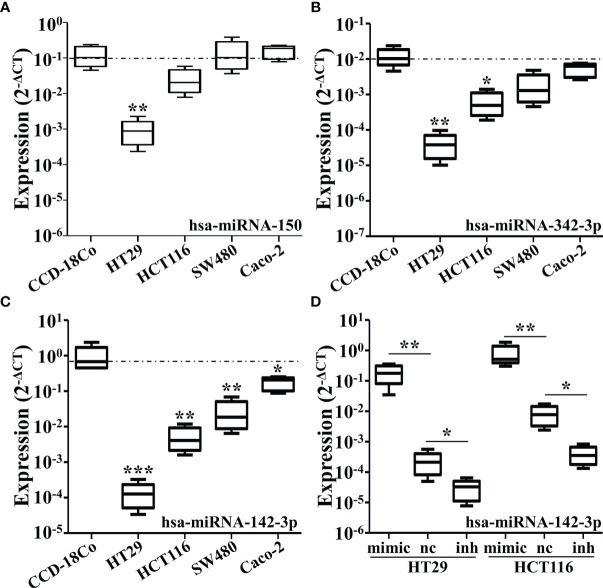
The expressions of miR-150, miR-342-3p, and miR-142-3p are down-regulated in various colorectal cancer cell lines. The qRT-PCR analysis revealed the expression levels of miR-150 **(A)**, miR-342-3p **(B)** and miR-142-3p **(C)** in several colon cancer cell lines including HT29, HCT116, SW480 and caco-2, compared with a human colon fibroblasts CCD-18Co. **(D)** The expression of miR-142-3p was be measured in the HT29 and HCT116 cells transduced with synthetic miRNA mimics. All data are shown as means ± SD. * and ** represent significant difference as p < 0.05 and 0.01 respectively.

To analyze the changes of miRNAs elicited by CRC, we firstly conducted the cluster analysis of miRNAs in the plasma of 10 diagnosed CRC patients and six healthy controls. Three miRNAs were found to be significantly down-regulated in CRC patients, miR-150, miR-342-3p and miR-142-3p (**Figure 1A**). The results were then verified by qRT-PCR (**Figures 1B–D**). The expression of these three miRNAs was also tested in four different CRC cell lines compared with CCD-18Co. It was shown that miR-150 was only shown to be reduced in HT29 cells and miR-342-3p was down-regulated in HT29 and HCT116 cells (**Figures 2A, B**), while miR-142-3p expression levels were significantly decreased in all of the four CRC cell lines compared to CCD-18Co cells (**Figure 2C**). To further explore the functions of miR-142-3p in CRC, miR-142-3p was overexpressed or repressed *via* transducing synthetic miRNA mimics or inhibitor in HT29 and HCT116 cells. The miR-142-3p expression levels were confirmed by qRT-PCR (**Figure 2D**).

### Overexpression of miR-142-3p Inhibits Cell Proliferation and Colony Formation of HT29 and HCT116 Cells

**Figure 3:**
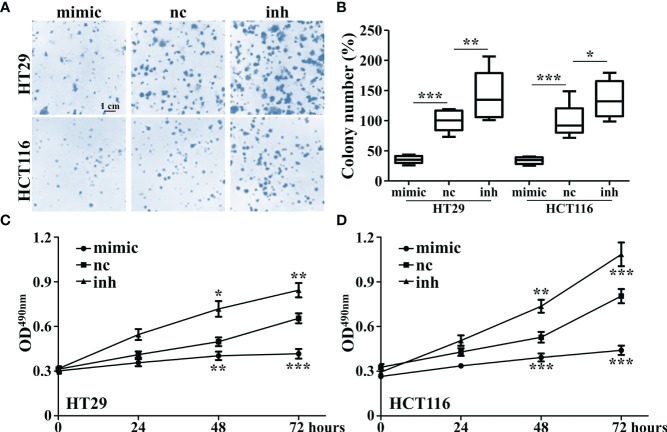
Overexpression of miR-142-3p inhibits cell proliferation and colony formation of HT29 and HCT116 cells. **(A)** Colon formation in colorectal cancer cell lines (HT29 and HCT116) after overexpression of miR-142. Scale bar, 1 cm. **(B)** Summary and statistics of **(A)**. **(C)** MTT assay was conducted on HT29 cells. **(D)** MTT assay was conducted on HCT116 cells. All data are shown as means ± SD. *, ** and *** represent significant difference as p < 0.05, 0.01, and 0.001, respectively.

It was found that overexpression of miR-142-3p led to a significant decrease of colony formation (**Figures 3A, B**) and cell proliferation (**Figure 3C, D**) of HT29 and HCT116 cells whereas inhibition of miR-142-3p showed adverse effects.

### MiR-142-3p Suppresses the Expression and Nuclear Accumulation of β-catenin by Directly Targeting CTNNB1

**Figure 4:**
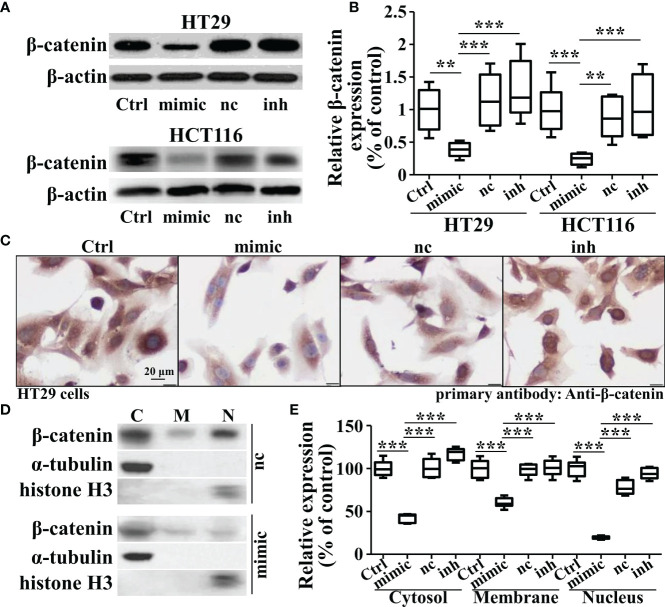
Overexpression of miRNA-142-3p inhibits the expression levels and patterns of β-catenin in colorectal cancer cells. **(A)** Representative Western blot and **(B)** Summary of the expression of β-catenin when overexpressing or repressing miRNA-142-3p levels. **(C)** Immunohistochemistry analysis of HT29 cells after overexpressing or repressing miRNA-142-3p. Scale bar, 20 μm. **(D)** Representative Western blot and **(E)** Summary of the cytosolic, membrane and nuclear expression β-catenin after cell fractionation. All data are shown as means ± SD. ** and *** represent significant difference as p < 0.01 and 0.001, respectively.

**Figure 5:**
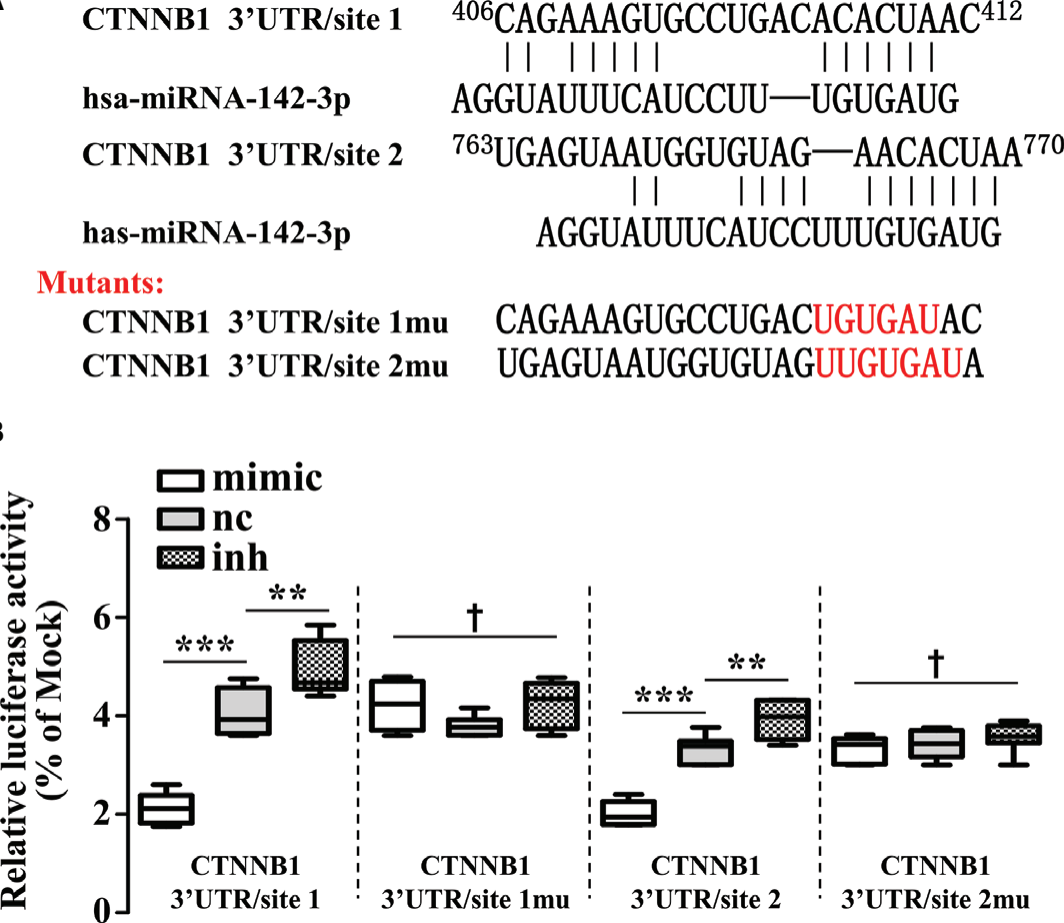
β-catenin is a direct target of miRNA-142-3p. **(A)** The two predicted binding sites and the mutations that disrupt the associations between miRNA-142-3p onto the β-catenin (CTNNB1) mRNA 3′-UTR. **(B)** The relative luciferase activity (FL/RL) was measured in HT29 cells after co-transfection of the β-catenin 3′-UTR or β-catenin 3′-UTR mutant luciferase constructs with either miRNA-142-3p mimics, NC, or inhibitors (100 nmol/L). All data are shown as means ± SD. *** represents significant difference with p < 0.001, while † represents p > 0.05. ** represents significant difference with p < 0.05.

As β-catenin is necessary for the activation of Wnt signaling, we measured the β-catenin expression in miR-142-3p overexpressed or repressed cells. Interestingly, miR-142-3p overexpression led to a dramatic decline of β-catenin expression while inhibition of its expression reversed this effect (**Figures 4A, B**). We further performed immunohistochemistry and found not only the expression but also the nuclear accumulation of β-catenin was inhibited after overexpression of miR-142-3p (**Figure 4C**). These observations were confirmed by testing the protein level of β-catenin after cell fractionation (**Figures 4D, E**). Next, we predicted two binding sites of miR-142-3p on β-catenin (CTNNB1). Two mutants of the predicted binding sites were constructed for luciferase assay. (**Figure 5A**). It was found that overexpression of miR-142-3p significantly repressed the transcriptional activity of CTNNB1 while inhibition of miR-142-3p promoted it (**Figure 5B**). However, these effects were not observed when mutants of CTNNB1 were transfected.

### MiR-142-3p Overexpression Represses Tumor Growth *In Vivo*

**Figure 6:**
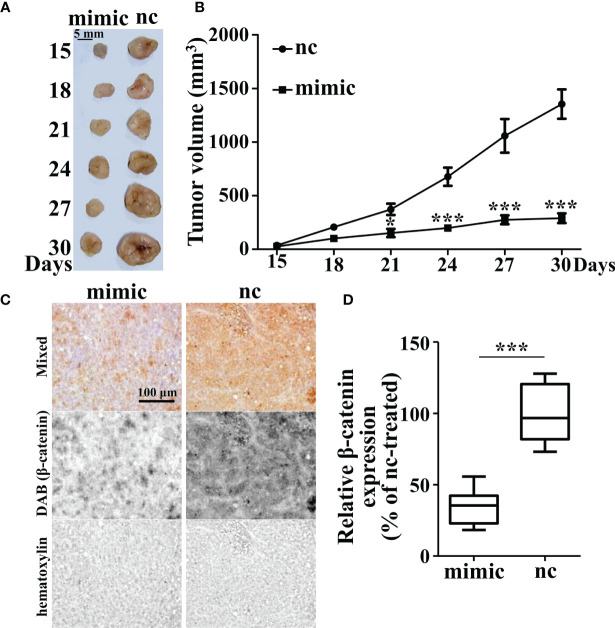
Tumor growth is inhibited by miR-142-3p overexpression in xenograft mice. **(A)** Tumor xenograft mice model. HT29-nc and HT29-miR-142-3p mimic cells were injected into the hindlimbs of nude mice (n = 6 at each time point). **(B)** Summary of tumor volume measurement. **(C)** Immunohistochemistry analysis of β-catenin expression in the tumor regions of both nc- and mimic-injected nude mice. Scale bar, 100 μm. **(D)** Summary of **(C)**. All data are shown as means ± SD. * and *** represent significant difference with p < 0.05 and 0.001, respectively.

To confirm the role of miR-142-3p in tumor growth, we further employed xenograft mice. Cells with miR-142-3p overexpression and its control cells were injected into the hindlimbs of nude mice and tumor volumes were measured and recorded. We observed that cells with miR-142-3p overexpression showed dramatically lower growth speed compared to its control cells (**Figures 6A, B**). Then, we evaluated the expression levels of β-catenin in the tumors and found that the formed tumors by cells with miR-142-3p overexpression showed a significantly lower level of β-catenin expression than those by control cells (**Figures 6C, D**).

## Discussion

A number of previous studies have implicated the association of miRNAs alterations in the plasma of healthy people and CRC patients. For example, a recent study reported that miR‐150, ‐142‐5p, and ‐29c were significantly lower in patients with higher T‐stage (10). Although this study demonstrated that it was difficult to apply these plasma miRNA levels to detect and diagnose CRC, another study demonstrated that the plasma levels of several miRNAs including miR-29a could be used as biomarkers for CRC recurrence after the operation (11). However, the present study mainly focused on whether the altered plasma miRNA also plays an important role in tumorigenesis and we aim to obtain deeper insights into its underlying mechanism. Here, we found that in comparison with healthy people, several miRNAs expression levels were reduced in the plasma of patients with CRC, including miR-324-3p, miR-142-3p, and miR150. We evaluated these results by qRT-PCR. It was found that miR-142-3p showed the most dramatic decrease in CRC patients compared to healthy people. Interestingly, we tested the expression of these miRNAs in four CRC cell lines. It was found that in all of the CRC cells, miR-142-3p expression showed a remarkable trend of decrease than CCD-18Co. It is possible that miR-142-3p could be produced and secreted from normal colon cells. Therefore, when its expression is decreased and tumorigenesis occurs, miR-142-3p is repressed and its plasma level is also reduced.

In fact, miR-142-3p has been widely reported in previous studies. However, most of its functions were demonstrated in stem cells, embryonic development or the immune system. For example, miR-142-3p was considered as an important factor in neutrophil development and hematopoietic stem cell formation and differentiation (12, 13). Recently, growing evidence has documented that miR-142-3p are associated with various cancers. In ovarian cancer cells, its expression level was dramatically reduced compared to normal ovarian epithelial cell line (14). Ectopic expression of miR-142-3p was reported to inhibit cell proliferation and chemoresistance by inhibiting SIRT1. In lung cancer, miR-142-3p was also downregulated and overexpression of it gave rise to a decrease of cell proliferation and increase of cell apoptosis, which leads to it as a tumor suppressor (15). In cervical cancer, gastric cancer and breast cancer, miR-142-3p showed a similar trend of change (16–18). Nevertheless, the results from several previous studies investigating the miR-142-3p expression in colon cancer tissues and cells showed controversial conclusions. Some of the studies demonstrated that CRC tissues and cell lines showed remarkably enhanced miR-142-3p levels (9, 19). It was reported that miR-142-3p activated Ras-related C3 botulinum toxin substrate 1 (RAC1) to promote cellular invasion (19). In contrast, there are also several studies showed in CRC tissues and cell lines, miR-142-3p was significantly decreased and miR-142-3p acted as a tumor suppressor in CRC (18, 20, 21). It was stated that overexpression of miR-142-3p strongly repressed tumorigenesis, which are consistent to our findings.

It was previously reported that in breast cancer stem cells miR-142-3p dramatically reduced the β-catenin levels (22). Another previous study found that miR-142-3p directly bound to the 3′ untranslated region (3′ UTR) of Ctnnb1 gene that encodes β-catenin (23). Repression of β-catenin by miR-142-3p was also observed in other tumors cells including glioma (24) and leiomyomas (25). It is known that β-catenin is a key modulator in Wnt signaling. Without Wnt ligands, cytoplasmic β-catenin proceed degradation after phosphorylated by a complex composing proteins including CK1 and GSK3)followed by ubiquitinated by β-TrCP (26). Wnt binds to its the receptors Frizzled (FZD) or low-density lipoprotein-related protein 5/6 (LRP5/6) to activate Wnt signaling by attracting the complex to the plasma membrane. Then, β-TrCP is dissociated from the complex and β-catenin proteins are released and translocated into the nucleus. Here, our results suggest that miR-142-3p directly binds to β-catenin. Overexpression of miR-142-3p repressed the protein levels and nucleus accumulation of β-catenin. These results provided evidence for miR-142-3p functioning an inhibitor of Wnt signaling. In addition, our findings are consistent with several recent reports (27), while APC is the target for miR-142-3p (28, 29). MiR-142-3p overexpression dramatically prevented tumor formation of HT-29 cells provided further evidence demonstrating miR-142-3p may serve as a tumor suppressor. This conclusion is not only drawn here but also was documented in previous studies on other cancer types such as testicular germ cell tumors (30) and non-small-cell lung carcinoma (15).

It is also quite interesting to mention that the inhibition of cell proliferation by miRNA-142-3p was exerted *via* a cell-cycle-dependent cell death (data not shown). However, this process of cell death was quite different from the conventional apoptosis which could be separated by a PI/Annexin-V double staining to be early and late phase. As a result, more experiments to elucidate the underlining mechanism of this phenomenon are conducting.

In this study, we screened the miRNA expression profiles in the plasma from healthy people or CRC patients and found that, compared with those of healthy people, there were three miRNAs levels that were significantly down-regulated in CRC patients. Among the three miRNAs, we identified that miR-142-3p expression was significantly lower in the CRC cell lines than that of the colon fibroblasts. Overexpression of miR-142-3p significantly diminished but the inhibitor of miR-142-3p promoted proliferation and colony formation of both HT29 and HCT116 cells. Our further investigations in the roles of miR-142-3p revealed that it could regulate the protein levels and nuclear accumulation of β-catenin. In conclusion, β-catenin is a direct target of miR-142-3p, which may be the underlying mechanism rendering miR-142-3p inhibiting tumor formation.

## Data Availability Statement

The raw data supporting the conclusions of this article will be made available by the authors, without undue reservation.

## Ethics Statement

The studies involving human participants were reviewed and approved by Changhai Hospital Ethics Committee. The patients/participants provided their written informed consent to participate in this study. The animal study was reviewed and approved by Changhai Hospital Ethics Committee.

## Author Contributions

Each of the authors contributed to the collection of the blood sample and to the drafting and editing of this manuscript. All authors contributed to the article and approved the submitted version.

## Funding

This work was supported by a grant from Science and Technology Commission Shanghai Municipality (17411951700), also by a grant from SMMU precision medicine program (2017JZ39) and a seed grant from the Changhai Hospital for young doctor (CH201703).

## Conflict of Interest

The authors declare that the research was conducted in the absence of any commercial or financial relationships that could be construed as a potential conflict of interest.
